# A Population-Based Model to Consider the Effect of Seasonal Variation on Serum 25(OH)D and Vitamin D Status

**DOI:** 10.1155/2015/168189

**Published:** 2015-09-01

**Authors:** Philippe Vuistiner, Valentin Rousson, Hugues Henry, Pierre Lescuyer, Olivier Boulat, Jean-Michel Gaspoz, Vincent Mooser, Peter Vollenweider, Gerard Waeber, Jacques Cornuz, Fred Paccaud, Murielle Bochud, Idris Guessous

**Affiliations:** ^1^Institute of Social and Preventive Medicine (IUMSP), University Hospital of Lausanne (CHUV), 1010 Lausanne, Switzerland; ^2^Biomedicine, University Hospital of Lausanne, 1011 Lausanne, Switzerland; ^3^Department of Genetic and Laboratory Medicine, Geneva University Hospitals, 1205 Geneva, Switzerland; ^4^Unit of Population Epidemiology, Division of Primary Care Medicine, Department of Community Medicine, Primary Care and Emergency Medicine, Geneva University Hospitals, 1205 Geneva, Switzerland; ^5^Department of Internal Medicine, Internal Medicine, University Hospital of Lausanne (CHUV), 1011 Lausanne, Switzerland; ^6^Department for Ambulatory Care and Community Medicine, University of Lausanne, 1011 Lausanne, Switzerland; ^7^Department of Epidemiology, Rollins School of Public Health, Emory University, Atlanta, GA 30322, USA

## Abstract

*Background*. We elaborated a model that predicts the centiles of the 25(OH)D distribution taking into account seasonal variation. *Methods*. Data from two Swiss population-based studies were used to generate (CoLaus) and validate (Bus Santé) the model. Serum 25(OH)D was measured by ultra high pressure LC-MS/MS and immunoassay. Linear regression models on square-root transformed 25(OH)D values were used to predict centiles of the 25(OH)D distribution. Distribution functions of the observations from the replication set predicted with the model were inspected to assess replication. *Results*. Overall, 4,912 and 2,537 Caucasians were included in original and replication sets, respectively. Mean (SD) 25(OH)D, age, BMI, and % of men were 47.5 (22.1) nmol/L, 49.8 (8.5) years, 25.6 (4.1) kg/m^2^, and 49.3% in the original study. The best model included gender, BMI, and sin-cos functions of measurement day. Sex- and BMI-specific 25(OH)D centile curves as a function of measurement date were generated. The model estimates any centile of the 25(OH)D distribution for given values of sex, BMI, and date and the quantile corresponding to a 25(OH)D measurement. *Conclusions*. We generated and validated centile curves of 25(OH)D in the general adult Caucasian population. These curves can help rank vitamin D centile independently of when 25(OH)D is measured.

## 1. Introduction

Vitamin D deficiency causes rickets in infants and osteomalacia in adults and contributes to osteoporotic fracture risk [1]. Molecular and epidemiological associations of vitamin D deficiency with other diseases such as cancers, cardiovascular disease, diabetes, depression, and multiple sclerosis have been reported [2–5]. Guessous recently discussed in this* journal* the relationship of vitamin D with extraskeletal complications [6].

Serum 25-hydroxyvitamin D (25(OH)D) is the major circulating form of vitamin D. It is currently the best marker of vitamin D status and is used by clinicians to determine a patient's vitamin D status [7]. While the definitions of adequate vitamin D levels differ, several reports suggested that vitamin D levels are low and vitamin D deficiency is very prevalent in general adult populations [8]. For example, in Switzerland (latitude 46°), the prevalence of vitamin D insufficiency (25(OH)D serum levels between 50 nmol/L and 75 nmol/L) and deficiency (<50 nmol/L) is 36% and 38%, respectively [9].

Most of the circulating vitamin D is synthesized from cholesterol following exposure to sunlight ultraviolet B (UVB), whereas a smaller amount is derived from diet and dietary supplements [2]. Because diet is a naturally low source of vitamin D, vitamin D levels are highly dependent on UVB exposure, even more in settings where food fortification policies are not implemented. Other factors such as body mass index (BMI), skin pigmentation, and geographical factors (e.g., latitude, altitude, and meteorological conditions) can influence circulating vitamin D levels [10].

The physiological mechanisms of seasonal variation in vitamin D levels are well described [11, 12]. In studies that collected seasonal data, the highest 25(OH)D levels were found in summer and autumn and lowest levels in winter and spring [9, 11, 13–16].

Gender, age, and fat mass seem to influence 25(OH)D seasonal variations [17]. Surprisingly, international and national guidelines on vitamin D measure, classification, and substitution do not include seasonal variation in their recommendations [18–20]. A major clinical implication is that seasonal variation of 25(OH)D can directly influence the diagnosis of vitamin D insufficiency and deficiency. Currently, a measure of serum 25(OH)D concentration performed at a given period of the year by a healthcare provider is used to reflect the patient's vitamin D status for the overall year although 25(OH)D levels could be meaningfully different. Two previous studies that assessed the effects of seasonal variation of 25(OH)D on the assessment of vitamin D status in New Zealand confirmed the need of seasonally adjusted thresholds for the diagnosis of vitamin D deficiency [17, 21]. Many people were predicted to have suboptimal 25(OH)D concentrations for a substantial proportion of the year, despite having apparently adequate concentrations at the time of testing [17]. We are not aware of such population-based analysis using European data. A recent study showed that, even with a comprehensive set of genetic, anthropometric, dietary, and lifestyle correlates, not more than 32.8% of the variation in 25(OH)D could be explained in the EPIC-Germany study and that food intake was only a weak predictor of 25(OH)D concentrations, while a strong seasonal fluctuation in 25(OH)D was shown [22].

Recognizing this, we used data from two large population-based Swiss studies to generate and replicate a simple tool that could help healthcare providers in interpreting a patient's vitamin D status independently of when the patient's 25(OH)D was measured, while taking into account age, gender, and BMI. The aim of the project was to find a model that predicts the centiles of the 25(OH)D distribution given age, sex, BMI, and date of the measurement. Such centile curves allow for more precision compared to usual recommendations since they provide adjusted predictions. Moreover, such model provides an insight of 25(OH)D levels at a different time point.

## 2. Materials and Methods

### 2.1. Original Dataset, the CoLaus Study

To generate the tool, we used the data from the CoLaus study (http://www.colaus.ch). The primary aim of the CoLaus study was to assess the prevalence of cardiovascular risk factors in the Caucasian population of Lausanne, Switzerland [23] (latitude 46°). The sampling procedure of the CoLaus study has been described elsewhere [23]. Briefly, the CoLaus study was population-based and included participants aged 35 to 75 years. The complete list of the Lausanne inhabitants aged 35–75 years (*n* = 56,694 in 2003) was provided by the population register of the city and served to sample the participants to the study. A simple, nonstratified random sample of 35% of the overall population was drawn, 8,121 people were eligible for interview, and 6,188 participants were finally included in the cohort (76.2%). Recruitment began in June 2003 and ended in May 2006. Data were collected by trained field interviewers using standardized questionnaires.

### 2.2. Replication Dataset, the Bus Santé Study

To replicate the tool, we used the data from the Bus Santé study. The Bus Santé study is an ongoing cross-sectional population-based study conducted in the State of Geneva (latitude 46°), Switzerland (http://epidemiologiepopulation.hug-ge.ch). Participants' recruitment has been described in detail previously [24]. Briefly, a representative sample of noninstitutionalized residents aged 20 and more years is selected independently throughout each year by using a standardized procedure using an annual residential list established by local government. The participation rate was 65%. To match with the CoLaus study period, subjects who participated in the Bus Santé study between 2005 and 2008 were included.

### 2.3. 25-Hydroxyvitamin D and Covariates

In both studies, venous blood samples were drawn after an overnight fast and stored at −80°C. In the CoLaus study, serum 25(OH)D was measured in a single center by an ultra high pressure liquid chromatography-tandem mass spectrometry (LC−MS/MS) system [25]. In the Bus Santé study, serum 25(OH)D was measured in a single center by using a commercial one-step immunoassay (Architect 25(OH)D, Abbott Architect i2000sr). The correlation between these two methods was determined using 125 serum samples; a Passing-Bablok regression was performed, resulting in an intercept of 8.71 95% CI [3.31; 13.55] a slope of 0.86 [0.76; 0.95] (see Figure S1 in Supplementary Material available online at http://dx.doi.org/10.1155/2015/168189), which indicates that LC-MS/MS measurements are slightly lower than Architect ones if values are higher than 62.07 nmol/L. For lower values, Architect measurements are higher.

In both studies, weight and height were measured using standardized procedures. BMI was defined as weight/height^2^ [kg/m^2^]. In the CoLaus study, information on personal medicines, including prescription and self-prescribed drugs, vitamin, mineral supplements, use of oral contraception, and hormonal replacement therapy, was collected. This information was not available in the Bus Santé study. The date of participation was recorded in both studies.

The CoLaus study and the Bus Santé study complied with the Declaration of Helsinki and were approved by the local Institutional Ethics Committees. All participants gave written informed consent.

### 2.4. Statistical Analysis

To predict the centiles of the 25(OH)D distribution, we applied linear regression models on the square-root transformed 25(OH)D values. Since normality and a constant variance (estimated via the residual variance from the regression) are approximately achieved on the transformed scale, it is possible to estimate any centile of the distribution. A back transformation then returns these centiles on the original scale. We considered various models to adjust for known determinants of 25(OH)D including gender, age, BMI, and the day when the measurement was made (via sine and cosine), including possible interactions between them, and we selected the best model according to Bayesian information criterion (BIC). In particular, the effect of age was negligible once adjusted for the other characteristics. Thus, age was not included in the final model, whereas some interactions between BMI and sex and between date of measurement and sex were included. Note that the BMI was included as −1/√BMI in our model to limit the influence of individuals with large BMI. This transformation has been used by other authors to transform variables related to obesity towards normality [26]. Because skin pigmentation influences vitamin D synthesis, analyses were limited to Caucasians. 25(OH)D levels are influenced by vitamin D therapy and supplementation. We therefore excluded participants from the original dataset who reported vitamin D therapy and supplementation (3.9% of the original dataset). In both datasets, only participants aged between 35 and 65 years with a BMI between 18 and 40 kg/m^2^ were kept, which reduced the sample sizes for the analyses. These BMI limits approximately correspond to percentiles 1% and 99%. Like elderly (i.e., >75 years old) participants, subjects with extreme BMI were excluded as they are more likely to suffer from an underlying disease. To account for the difference between the two 25(OH)D measurement methods, we transformed the 25(OH)D values from the Bus Santé study according to the Passing-Bablok regression analysis (on the square-root transformed 25(OH)D values) performed on 125 random serums analyzed with the two methods.

The best model based on BIC was given as follows:

(1) $$\begin{array}{c} \sqrt{25\left(\text{OH}\right)\text{D}}=\beta_{0}+\beta_{1}\sin\left(\frac{2\pi \left(day-1\right)}{365}\right)+\beta_{2}\cos\left(\frac{2\pi \left(day-1\right)}{365}\right)+\beta_{3}\sin\left(\frac{4\pi \left(day-1\right)}{365}\right)+\beta_{4}\cos\left(\frac{4\pi \left(day-1\right)}{365}\right)+\beta_{5}t\left(BMI\right)+\beta_{6}t{\left(BMI\right)}^{2}+\beta_{7}I\left(male\right)I\left(BMI<25\right){\left(t\left(BMI\right)-t\left(25\right)\right)}^{2}+\beta_{8}I\left(female\right)\sin\left(\frac{2\pi \left(day-1\right)}{365}\right)+\beta_{9}I\left(female\right)\cos\left(\frac{2\pi \left(day-1\right)}{365}\right). \end{array}$$

BMI was transformed using an inverse square-root function *t*(*x*) = −1/√*x*.

Model's coefficients were as follows: *β*_0_ = −2.754 (*P* value = 0.295), *β*_1_ = −1.077 (*P* value < 0.0001), *β*_2_ = −0.756 (*P* value < 0.0001), *β*_3_ = 0.188 (*P* value < 0.0001), *β*_4_ = 0.025 (*P* value = 0.368), *β*_5_ = −81.08 (*P* value = 0.002), *β*_6_ = −165.6 (*P* value = 0.014), *β*_7_ = −1174 (*P* value ≤ 0.0001), *β*_8_ = 0.218 (*P* value < 0.0001), and *β*_9_ = 0.164 (*P* value = 0.003). The model explained 29% of the variance.

Of note, the equation *β*_1_sin⁡(2*π*(day − 1)/365) + *β*_2_cos⁡ (2*π*(day − 1)/365) is equivalent to *β*_1_′sin⁡ (2*π*(day − 1)/365 + *β*_2_′) that has been used in previous work by Bolland et al. [21] with *β*_1_^  ^ = *β*_1_′cos ⁡(*β*_2_′) and *β*_2_ = *β*_1_′sin⁡ (*β*_2_′). In our model, we added two supplementary sine-cosine terms that allow the time between minimum and maximum levels to be different from 6 months. The lowest predicted values were at mid-March and the highest at mid-August.

We then used the standard deviation (SD) of the residuals (*σ*_*ϵ*_ = 1.36) to predict all centiles of the 25(OH)D distribution given fixed values for sex, BMI, and day of measurement. The *α* centile is given as $\hat{y}+z_{\alpha }\sigma_{\epsilon }$, where $\hat{y}$ is the fitted value and *z*_*α*_ is the *α* quantile of a standard normal distribution. The square of this centile returns the value on the original scale.

To validate the model, we accounted for the fact that some participants in the Bus Santé had vitamin D supplementation (without knowing which ones). We refitted our regression model above on the CoLaus participants with and without vitamin D supplementation (*N* = 5,066) and used these model coefficients to estimate the 25(OH)D values in the Bus Santé study. For each observation, we estimated the corresponding quantile. Histogram and empirical cumulative distribution function of the quantiles of the Bus Santé observations predicted with the model based on the CoLaus data were inspected. Quantiles should be approximatively uniformly distributed between 0 and 1 if the model is correct.

## 3. Results

Overall, 4,912 subjects were included in the original dataset (CoLaus study). Participants' characteristics are shown in Table 1. The mean (SD) age and mean BMI were 49.8 (8.5) years and 25.6 (4.1) kg/m^2^, respectively. Fifty % (2491/4912) of the participants were women, 14.3% (703/4912) were obese (i.e., BMI ≥ 30), and 10.1% (251/2491) of women used oral contraceptive or hormonal replacement. The number (%) of participants by month varied from 268 (5.5%) to 526 (10.7%) (see Table S1) with fewer people measured during the summer months (May to August).

The mean (SD) 25(OH)D level was 47.6 (22.1) nmol/L (Table 1). The mean 25(OH)D levels differed by season, gender, and BMI (see Table S2). Overall, 25(OH)D levels were <50 nmol/L and <75 nmol/L in 57.5% and 31.1% of the participants.

**Figure 1:**
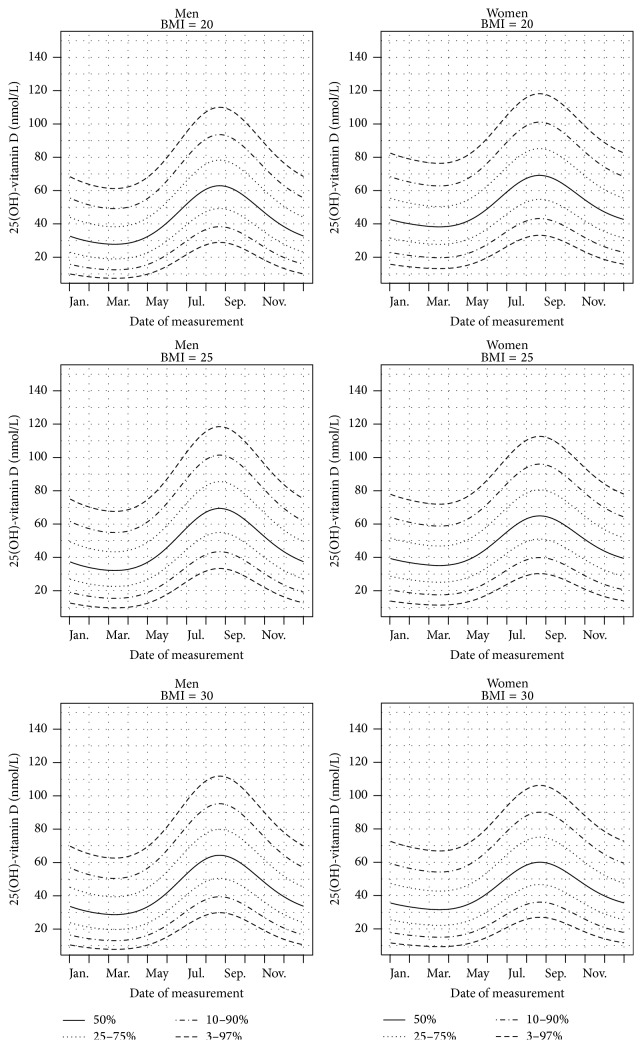
Quantiles of 25(OH)D according to the date of measurement (horizontal units expressed in month of measurements), for men and women with different BMI values (20, 25, and 30).

Centile curves for 25(OH)D predicted with the multivariate model as a function of date of measurement for men and women with a fixed value of BMI are illustrated in Figure 1 expressed as a function of month of measurements and in Figure S2 expressed as a function of week of measurements. The interaction between gender and BMI appears clearly when looking at the quantiles predicted as a function of the BMI for a given day of measurement, as presented in Figure S3. August 22 is found to be the day with the highest predicted 25(OH)D values, for both men and women. The lowest predicted levels are on March 11 for men and on March 18 for women. This difference is due to the sex-day interaction.

Given values for sex, BMI, and day of measurement, it is possible to estimate any centile of the 25(OH)D distribution. For example, the median 25(OH)D value measured on August 22 of a woman with a BMI of 20 is 69.1 nmol/L. Another important aspect of this model is that it can be used to estimate the quantile corresponding to a 25(OH)D measurement. For example, a man with a BMI of 25 and a 25(OH)D level of 60 nmol/L measured on January 1 will be on the 88.6% quantile.

Our model fitted the data pretty well, as can be seen in Figure S4, where the histogram and the empirical cumulative distribution function of the quantiles predicted by the model were almost those of a uniform distribution.

### 3.1. Replication

Overall, 2,537 subjects were included in the replication dataset (Bus Santé study). Participants' characteristics are shown in Table 1. The replication sample differed from the original in several ways. The number (%) of participants by month varied from 59 (2.3%) to 298 (11.8%) (see Table S1).

The uncorrected mean 25(OH)D values were higher in the Bus Santé than in CoLaus. The mean (SD) 25(OH)D level was 50.5 (19.9) nmol/L. The mean 25(OH)D levels differed by season, gender, and BMI (see Table S2). After correction for difference in methods used, the mean (SD) 25(OH)D value in the Bus Santé was similar to the mean 25(OH)D in CoLaus (48.6 nmol/L versus 48.1 nmol/L, *P* value = 0.39, see Table 1). Histogram and empirical cumulative distribution function of the quantiles of the Bus Santé observations predicted with the model based on the CoLaus were very close to uniformity suggesting that the model is correct (see Figure S5). To check the validity of our prediction, the predicted percentage of people under a given quantile can be considered. For example, 12.1% of participants have a predicted 25(OH)D value under the 10% centile, or 4.1% are predicted under the 3% centile. These proportions are slightly overestimated (as shown in the histogram in Figure S5) but close to correct though. The highest difference between measured and predicted quantiles is given by the Kolmogorov-Smirnov test statistic, which was equal to 0.036 (95% CI [0.026; 0.054]).

## 4. Discussion

Using two large Swiss population-based studies, we generated and validated centile curves of 25(OH)D in the general adult population. These curves can help healthcare providers in interpreting a patient's vitamin D status independently of when the patient's 25(OH)D was measured. We provided gender- and BMI-specific 25(OH)D centile curves as 25(OH)D level is influenced by these factors. We developed a free online tool available at http://www.iumsp.ch/charts/select_graph.htm that can be used to determine where a patient fits compared to a reference population at a given time.

Given the growing evidence of associations of low vitamin D levels with several chronic diseases, determining vitamin D status has gained increasing importance [27], especially in settings such as in Switzerland where no vitamin D fortification policy is implemented. Efforts to find a sufficiently predictive model for vitamin D status without using information on 25(OH)D level have failed and currently there is no substitute for 25(OH)D testing [28]. While the best determinant of vitamin D status is the serum concentration of 25(OH)D, this parameter fluctuates with season and not considering season-specific 25(OH)D levels can mislead the diagnosis of vitamin D status. Two studies conducted in New Zealand previously assessed the effects of seasonal variation of 25(OH)D on diagnosis of vitamin D sufficiency [17, 21]. In the most recent one, Bolland et al. used the data from postmenopausal women (*N* = 1,606) and from a small sample (*N* = 378) of middle-aged and older men and found that many people were predicted to have suboptimal 25(OH)D concentrations for a substantial proportion of the year, despite having apparently adequate concentrations at the time of testing [21]. Results observed in New Zealand are however not directly transposable to European settings given that 25(OH)D levels are influenced by latitude and sunshine duration. Brown et al. proposed an interesting mathematical model of 25(OH)D serum using information on food, supplements, sun, and 25(OH)D levels [29]. Yet, information on 25(OH)D levels dated from 1998, and compared to this present study, sample size was smaller and BMI was not taken into account. Thus, our work provides information using more recent data collected in a larger study sample from Switzerland, a country situated in the center of Europe.

Furthermore, the number of large population-based studies reporting seasonal data is limited [8, 15, 16, 30], and we found no study that reported centile curves. Centile curves present several advantages. Centile curves are routinely used in clinical medicine to detect extreme values of a measurement of interest [31, 32]. Data used to construct the curves are representative of the reference population. Centile curves can take into consideration major factors (e.g., gender, BMI, and date of measurement) that influence the distribution. In line with a previous Swiss population-based study [9], age was not independently associated with 25(OH)D. The lack of an independent association might be explained, at least in part, by the upper age limit of the participants (i.e., 75 years) included in the CoLaus and Bus Santé studies and/or by the adjustment for BMI. Age-related vitamin D deficiency has been essentially reported among elderly subjects; cutaneous production of vitamin D is decreased [33] and exposure to sunshine through outdoor activities reduced in elderly subjects [34]. The effect of age on vitamin D deficiency among subjects younger than 75 years might be not strong enough to be captured after adjustment for gender, BMI, and day of measurement. BMI frequently increases with age [35] and there are several mechanisms by which vitamin D levels could be inversely associated with BMI including the decrease of outdoor physical activity, the inadequate diet, and the sequestration of vitamin D in the cutaneous fat among people with overweight or obesity [36]. Our results suggest that BMI and not age is worth considering when interpreting seasonal variation of 25(OH)D. Nevertheless, only adult participants between 35 and 65 years were included. When considering children, adolescents, and elderly people, the age association might be different from what was observed in the present study.

While not considering season-specific 25(OH)D levels biases the assessment of vitamin D status, the benefit of maintaining optimal 25(OH)D levels throughout the seasons remained to be demonstrated. There is some evidence suggesting that cycles of suboptimal level of vitamin D throughout the year are harmful for bone health [37–39], but such evidence for other health outcomes is little [11, 40]. The observation that not only the parathyroid hormone but also the active form of vitamin D (i.e., 1,25(OH)2D) parallels 25(OH)D seasonal variation [41] suggests that variation, if meaningful enough, can influence health outcomes. In general, any period of vitamin D insufficiency is undesirable [17, 21, 42].

Finally, given the cost of 25(OH)D testing and the evidence of overtesting, some insurance and regions have altered their policies to restrict 25(OH)D testing [28, 43]. In the absence of vitamin D supplementation, our simple tool, which relies on a single 25(OH)D test, could potentially decrease healthcare cost by limiting the number of 25(OH)D tests performed for a given patient throughout a year to determine vitamin D status.

### 4.1. Strengths and Limitations

When interpreting the findings of this study, one has to keep in mind its limitations. First, our results are representative of general and unselected adult populations in Switzerland. Because vitamin D levels have been shown to vary between populations, the centile curves presented in this report are not generalizable to populations that differ from our source population in terms of both individual- (e.g., age, ethnicity) and contextual-determinants of vitamin D levels (e.g., food fortification policy). Of note, these results are latitude-specific as both studies were conducted at the same latitude. When determinants of vitamin D levels are similar, our results can potentially be extrapolated to European countries within the same range of latitude (45°–50°) such as France, Austria, the southern part of Germany, the northern part of Italy, and some eastern European countries. For other countries, our method could be replicated when population-based data are available. Second, we relied on a single measure of vitamin D per participant. Third, as previous studies [17, 21], if we want to predict future values of an individual along the year given a measurement made on a given day, we have to assume that each participant's 25(OH)D concentrations throughout the year would follow a curve similar to that of the overall population; that is, the correlation between two repeated measurements made on the same subjects in different seasons would be perfect (1.0). To be able to affine such individual predictions taking into account that this correlation is not perfect, data from a longitudinal analysis would be necessary. While our model was built to fit the seasonal variation of 25(OH)D at the population level, seasonal variation at the individual level can differ, notably in individuals who differ meaningfully from the study population in terms of vitamin D levels determinants. Fourth, we lack information on vitamin D therapy or supplementation in the replication dataset. We corrected the replication dataset 25(OH)D levels and centile curves using original dataset derived correction factor. Moreover, the measurement methods were different between the two datasets.

Another limitation could be the possible time trends of 25(OH)D levels from the data collection up to now. Samples were collected almost 10 years ago and we cannot exclude that population-averaged levels have changed. Yet, trends analyses of 25(OH)D levels in the Swiss population suggest that levels have been stable [9].

Our study has several strengths. To generate centile curves, we used standard reference 25(OH)D method of measurement (LC-MS/MS). We replicated our findings using a similar yet different population and a standard commercial immunoassay. The city of Lausanne (CoLaus study) and the state of Geneva (Bus Santé study) are located 37 miles away on the same latitude. Reference populations are ideally composed of individuals assumed to be “normal.” We used data from noninstitutionalized residents randomly selected to participate in population-based studies as reference populations. Compared to other study designs (clinical trials, hospital data, and institutions data), this approach mitigates the risk of having unrepresentative populations. Finally, we did not rely on healthcare-related administrative data, which are subject to bias including seasonal testing bias [28].

In conclusion, we used a centile curves approach, which is well known with growth charts, to generate and validate a model available as a free online tool that could help in interpreting vitamin D status independently of when 25(OH)D is measured.

## Supplementary Material

The supplementary material displays the number (%) of participants included in the analysis, by month (Table S1), the mean (SD) 25(OH)D levels for different period of the year and categories of participants (Table S2), a scatter plot with Passing & Bablok Fit (Figure S1), quantiles of 25(OH)D according to the date of measurement (horizontal units expressed in week of measurements), for men and women with a BMI equal to the median value (Figure S2), quantiles of 25(OH)D predicted as a function of BMI for a given day of measurement (August 22nd was found to be the day with the highest predicted 25(OH)D values, for both men and women) (Figure S3), and histogram and empirical cumulative distribution function (ECDF) of the quantiles predicted with the model, using the data from CoLaus (N=4,912) (Figure S4).

## Figures and Tables

**Table 1:** Participants' characteristics for CoLaus and Bus Santé studies.

	CoLaus study			Bus Santé study (*N* = 2,537)	*P* value^1^	*P* value^2^	*P* value^3^
	Participants with vitamin D supplementation excluded (*N* = 4,912)	Participants with vitamin D supplementation (*N* = 154)	Participants with and without vitamin D supplementation (*N* = 5,066)				
Age, mean (SD)	49.77 (8.54)	55.97 (7.30)	49.96 (8.57)	53.94 (6.73)	<0.001	<0.001	<0.001
Male gender, *N* (%)	2421 (49.3)	15 (9.7)	2436 (48.1)	1165 (45.9)	<0.001	0.006	0.075
Height, cm, mean (SD)	169.27 (9.26)	164.59 (7.52)	169.13 (9.24)	168.66 (9.40)	<0.001	0.008	0.041
Weight, kg, mean (SD)	73.59 (14.19)	64.73 (11.96)	73.32 (14.21)	72.30 (14.09)	<0.001	<0.001	0.003
BMI, kg/m^2^, mean (SD)	25.61 (4.12)	23.85 (3.67)	25.56 (4.12)	25.32 (3.94)	<0.001	0.003	0.015
25(OH)D, nmol/L, mean (SD)	47.58 (22.10)	66.05 (21.02)	48.14 (22.29)	48.62^*∗*^ (23.75)	<0.001	0.061	0.389
25(OH)D < 50 nmol/L, *N* (%)	2827 (57.5)	35 (22.7)	2862 (56.5)	1442^*∗*^ (56.8)	<0.001	0.555	0.775
25(OH)D 50–75 nmol/L, *N* (%)	1528 (31.1)	64 (41.6)	1592 (31.4)	776^*∗*^ (30.6)	0.006	0.645	0.457

^1^ *P* value comparing CoLaus study participants with and without vitamin D supplementation.

^2^ *P* value comparing CoLaus study participants without vitamin D supplementation and Bus Santé.

^3^ *P* value comparing CoLaus study (participants with and without vitamin D supplementation) and Bus Santé.

^*∗*^Based on corrected 25(OH)D values.
