# Supplementary material for: A Population-Based Model to Consider the Effect of Seasonal Variation on Serum 25(OH)D and Vitamin D Status
Source: Biomed Res Int. 2015 Sep 1;2015:168189. doi: 10.1155/2015/168189 (PMC4569755; doi:10.1155/2015/168189)

Table S1. Number (%) of participants included in the analysis, by month

	CoLaus		Bus Santé study (N=2537)
	Participants with vitamin D supplementation excluded (N=4912)	*Participants with and without vitamin D supplementation (N=5066)*	
January	415 (8.5)	428 (8.5)	116 (4.6)
February	422 (8.6)	435 (8.6)	202 (8.0)
March	517 (10.5)	530 (10.5)	285 (11.2)
April	386 (7.9)	401 (7.9)	274 (10.8)
May	268 (5.5)	274 (5.4)	298 (11.8)
June	333 (6.8)	340 (6.7)	240 (9.5)
July	359 (7.3)	370 (7.3)	205 (8.1)
August	351 (7.2)	367 (7.2)	59 (2.3)
September	395 (8.0)	407 (8.0)	161 (6.4)
October	420 (8.6)	435 (8.6)	256 (10.1)
November	526 (10.7)	546 (10.8)	276 (10.9)
December	520 (10.6)	533 (10.5)	165 (6.5)

Table S2. Mean (SD) 25(OH)D levels for different period of the year and categories of participants

	**CoLaus study**				**Bus Santé (N=2537)**			
	Participants with vitamin D supplementation excluded (N=4912)		*Participants with and without vitamin D supplementation (N=5066)*		*Uncorrected 25(OH)D values*		*Corrected 25(OH)D values*	
	Men	Women	Men	Women	Men	Women	Men	Women
Spring	42.0 (17.9)	44.4 (19.3)	42.0 (17.8)	45.2 (19.8)	47.9 (18.6)	48.5 (18.7)	45.5 (22.3)	46.3 (23.5)
Summer	65.7 (21.6)	63.5 (20.5)	65.7 (21.5)	64.2 (20.7)	62.3 (20.1)	59.9 (19.8)	62.7 (24.5)	59.8 (24.0)
Autumn	47.6 (19.1)	49.2 (20.9)	47.7 (19.1)	50.0 (21.1)	51.4 (18.1)	53.9 (20.1)	49.6 (21.6)	52.7 (24.0)
Winter	33.2 (16.8)	38.3 (19.0)	33.3 (16.8)	39.8 (20.2)	40.5 (17.5)	45.6 (19.9)	36.9 (20.5)	43.0 (23.5)
BMI<25 kg/m^2	48.8 (23.4)	51.0 (22.6)	48.9 (23.3)	52.1 (23.0)	53.0 (22.2)	54.2 (20.7)	51.7 (26.7)	53.1 (24.7)
25≤BMI<30 kg/m^2	46.9 (21.5)	45.6 (20.1)	46.9 (21.5)	46.3 (20.3)	48.9 (18.1)	47.6 (18.3)	46.7 (21.6)	45.1 (21.5)
BMI≥30 kg/m^2	42.9 (21.4)	40.6 (19.6)	43.0 (21.4)	41.3 (20.0)	44.5 (15.6)	40.6 (16.1)	41.4 (18.3)	37.0 (18.6)
Age<45	47.1	48.2	47.1	48.2	53.3	51.6	51.9	50.1

years	(21.5)	(22.3)	(21.5)	(22.3)	(19.4)	(22.8)	(23.5)	(27.3)
45≤Age<55 years	46.3 (22.3)	47.2 (21.0)	46.3 (22.2)	48.2 (21.5)	48.7 (19.4)	49.4 (19.1)	46.6 (23.1)	47.4 (22.6)
Age≥55 years	47.8 (23.3)	49.0 (22.2)	47.9 (23.2)	50.9 (22.9)	50.0 (20.0)	52.1 (20.1)	48.1 (23.9)	50.5 (23.9)

Figure S1. Scatter Plot with Passing & Bablok Fit.

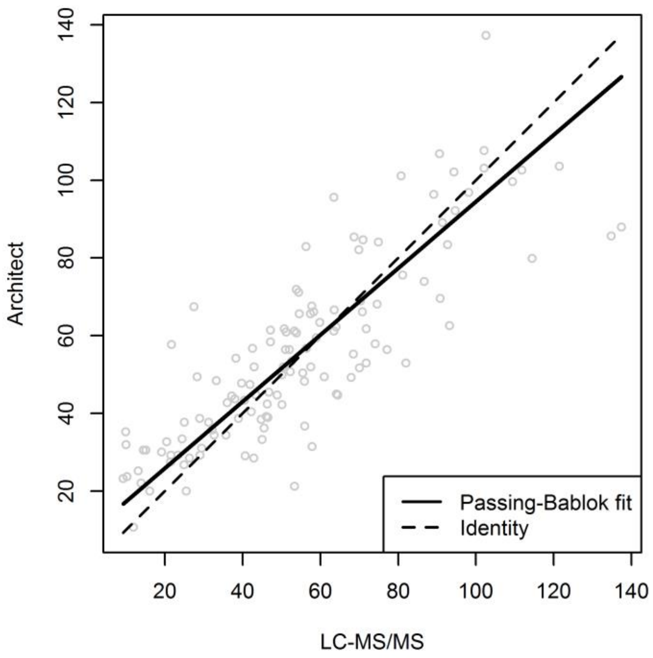

Figure S2. Quantiles of 25(OH)D according to the date of measurement (horizontal units expressed in week of measurements), for men and women with a BMI equal to the median value

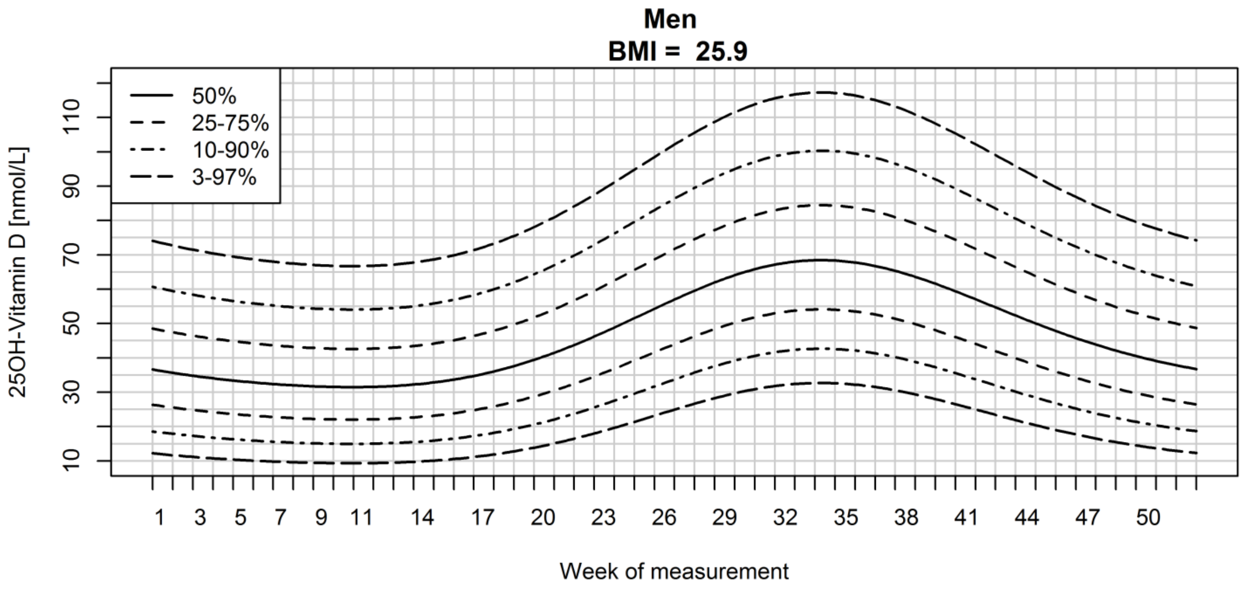

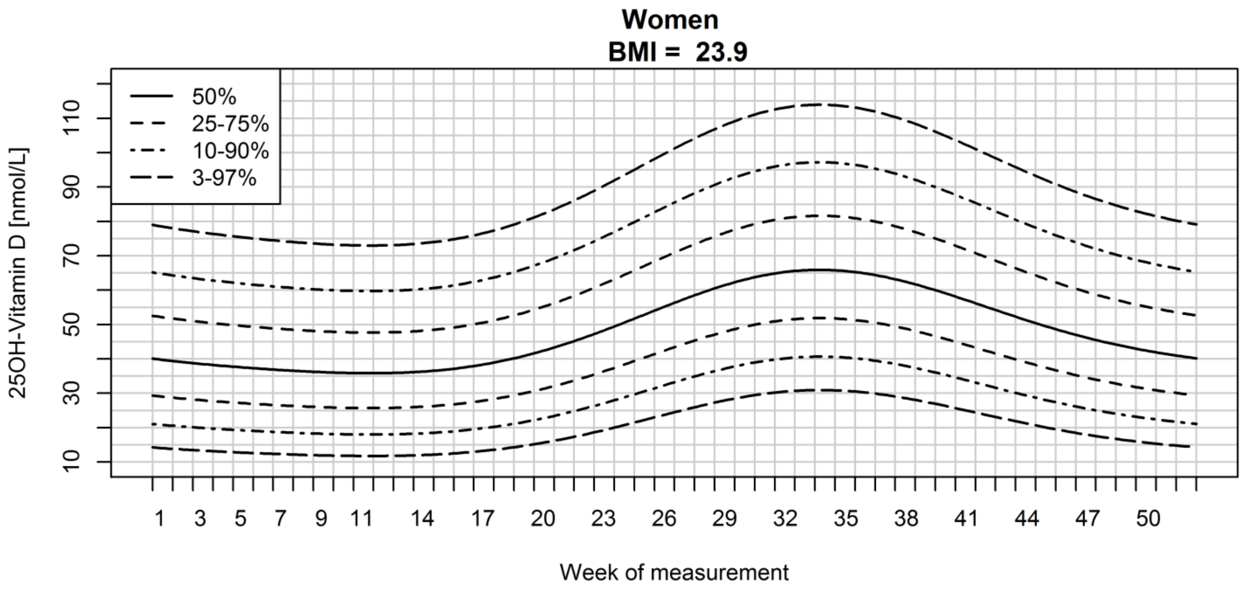

Figure S3. Quantiles of 25(OH)D predicted as a function of BMI for a given day of measurement (August 22nd was found to be the day with the highest predicted 25(OH)D values, for both men and women).

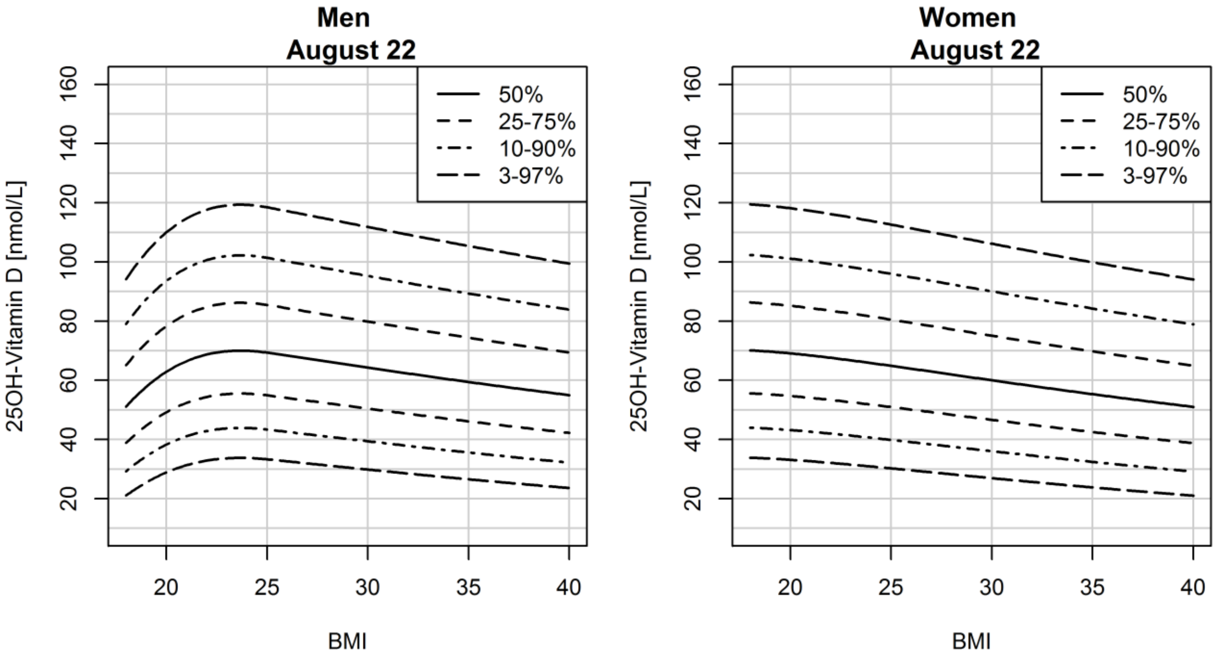

Figure S4. Histogram and empirical cumulative distribution function (ECDF) of the quantiles predicted with the model, using the data from CoLaus (N=4,912).

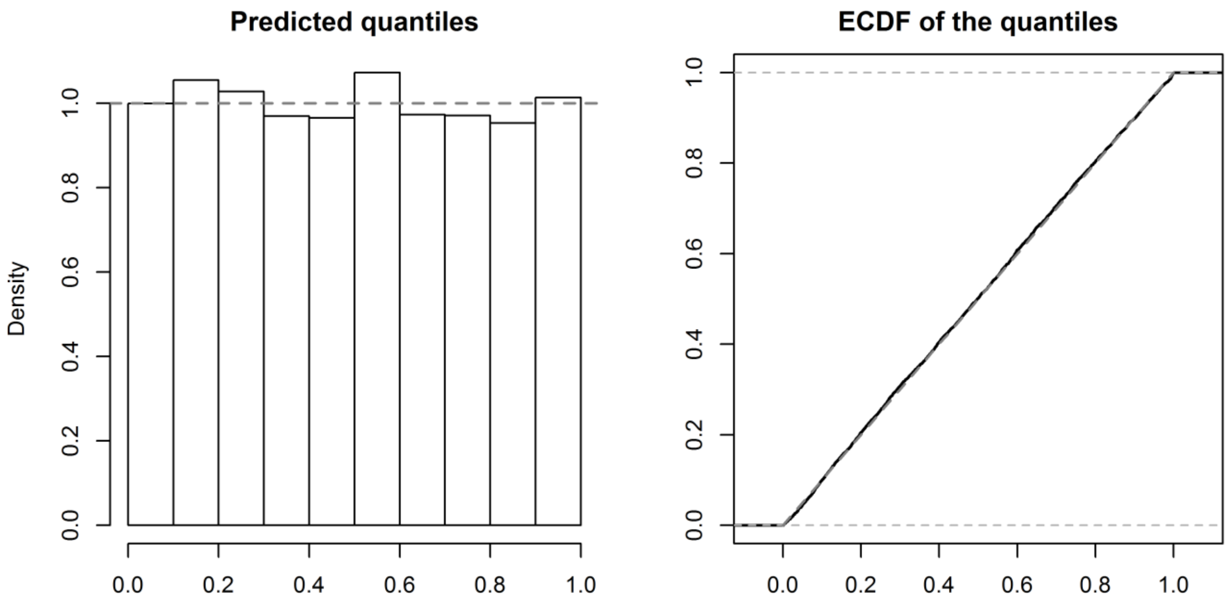

Figure S5. Histogram and empirical cumulative distribution function (ECDF) of the quantiles predicted with the model, using the data from Bus Santé (N=2,537).

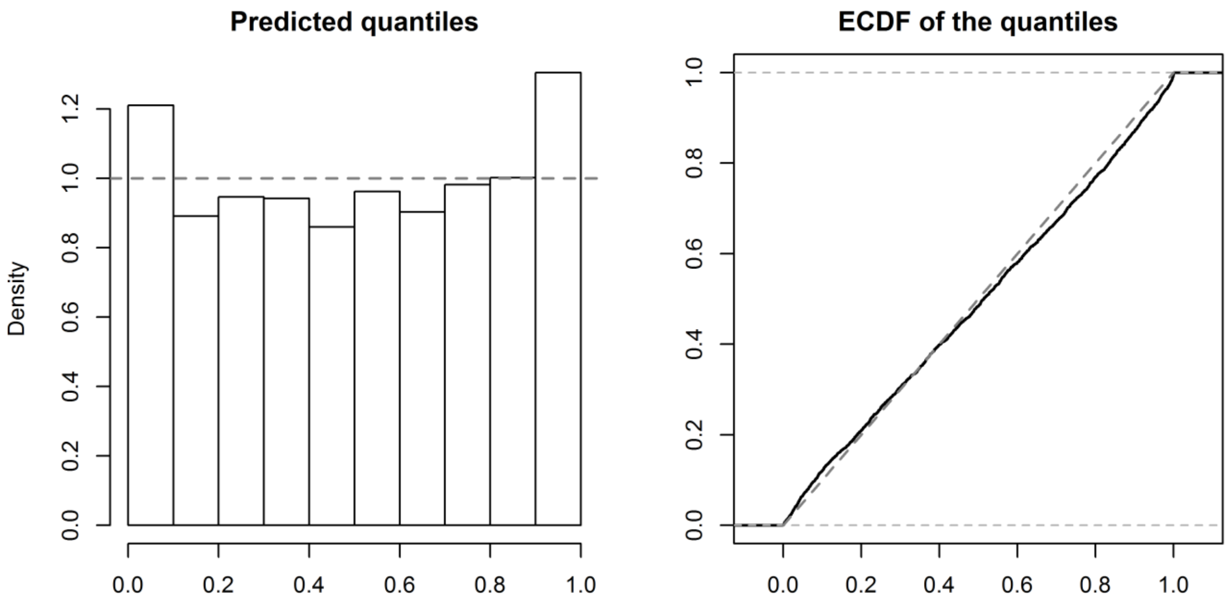

Supplement: Supplementary file 1 — The supplementary material displays the number (%) of participants included in the analysis, by month (Table S1), the mean (SD) 25(OH)D levels for different period of the year and categories of participants (Table S2), a scatter plot with Passing & Bablok Fit (Figure S1), quantiles of 25(OH)D according to the date of measurement (horizontal units expressed in week of measurements), for men and women with a BMI equal to the median value (Figure S2), quantiles of 25(OH)D predicted as a function of BMI for a given day of measurement (August 22nd was found to be the day with the highest predicted 25(OH)D values, for both men and women) (Figure S3), and histogram and empirical cumulative distribution function (ECDF) of the quantiles predicted with the model, using the data from CoLaus (N=4,912) (Figure S4). [file 168189.f1.pdf]
